# Supplementary material for: Helminth infection dynamics in rehabilitating Javan slow lorises are driven by time since deworming rather than host traits
Source: Int J Parasitol Parasites Wildl. 2026 May 27;30:101242. doi: 10.1016/j.ijppaw.2026.101242 (PMC13241643; doi:10.1016/j.ijppaw.2026.101242)
Supplement: Multimedia component 1 [file mmc1.docx]

**Helminth infection dynamics in rehabilitating Javan slow lorises are driven by time since deworming rather than host traits**

Abdullah Langgeng^1*^, Marie Sigaud^2^, Wendi Prameswari^3^, Nur Purba Priambada^3^, Puji Rianti^4,5^, Richard Moore^3^, Andrew J.J. MacIntosh^6^ Ikki Matsuda^1,7,8^,

*^1^ Wildlife Research Center of Kyoto University, 2‑24, Tanaka‑Sekiden‑Cho, Sakyo, Kyoto 606‑8203, Japan*

*^2^Muséum national d'Histoire naturelle Paris, France*

*^3^Yayasan Inisiasi Alam Rehabilitasi Indonesia, Bogor, West Java, Indonesia*

*^4^Department of Biology, Faculty of Mathematics and Science, IPB University, Bogor, West Java, Indonesia*

*^5^Program of Bio-conservation, Primate Research Center, IPB University, Bogor, West Java, Indonesia*

*^6^ Wilder Institute, Calgary, Canada*

*^7^Chubu Institute for Advanced Studies, Chubu University, 1200, Matsumoto-cho, Kasugai-shi, Aichi 487-8501, Japan*

*^8^ Institute for Tropical Biology and Conservation, Universiti Malaysia Sabah, Kota Kinabalu, Malaysia*

*correspondence to: abdullahlanggeng@gmail.com

AL: [abdullahlanggeng@gmail.com](mailto:abdullahlanggeng@gmail.com): ORCiD: [0000-0003-4896-6704](https://orcid.org/0000-0003-4896-6704)

MS: marie.sigaud@mnhn.fr: ORCiD: 0000-0002-6958-7239

WP: wendi@yiari.or.id; ORCiD : 0009-0003-0226-2135

NPP: purbo@yiari.or.id: ORCiD: 0009-0003-5228-7541

PR: pujirianti@apps.ipb.ac.id: ORCiD: 0000-0002-0898-4310

RM: richard@yiari.or.id; ORCiD: 0009-0003-8804 9211

IM: [matsuda.ikki.5a@kyoto-u.ac.jp](mailto:matsuda.ikki.5a@kyoto-u.ac.jp); ORCiD: 0000-0002-0861-7801

AJJM: [andrew.j.j.macintosh@gmail.com](mailto:andrew.j.j.macintosh@gmail.com): ORCiD: 0000-0002-9136-7099

Table S1. Study subject and fecal sampling summary for rehabilitating *Nycticebus javanicus* included in this study. Shown are individual identity, sex, release candidacy status (candidate or non-candidate), housing condition (group or solitary), year of admission to the YIARI rehabilitation center, and the number of fecal samples collected prior to deworming or for non-release candidates (“Pre/non”) and during the soft-release period for release candidates (“Soft”). All individuals were adults at the time of sampling.

| **ID** | **Sex** | **Release candidacy status** | **Housing condition** | **Year admitted to YIARI** | **Number of fecal samples collected** | |
| --- | --- | --- | --- | --- | --- | --- |
|  |  |  |  |  | Pre/non | Soft |
| **JS01** | Female | Candidate | Solitary | 2022 | 13 | 1 |
| **JS02** | Female | Candidate | Group | 2024 | 6 | 0 |
| **JS03** | Female | Candidate | Solitary | 2023 | 12 | 1 |
| **JS04** | Male | Candidate | Solitary | 2024 | 11 | 1 |
| **JS05** | Female | Candidate | Group | 2022 | 8 | 0 |
| **JS06** | Female | Candidate | Group | 2024 | 10 | 1 |
| **JS07** | Female | Candidate | Solitary | 2023 | 10 | 1 |
| **JS08** | Female | Candidate | Group | 2024 | 6 | 1 |
| **JS09** | Male | Candidate | Solitary | 2024 | 12 | 1 |
| **JS10** | Male | Candidate | Group | 2024 | 5 | 0 |
| **JS11** | Female | Non-candidate | Solitary | 2016 | 6 | 0 |
| **JS12** | Female | Non-candidate | Solitary | 2012 | 5 | 0 |
| **JS13** | Male | Non-candidate | Solitary | 2017 | 6 | 0 |
| **JS14** | Female | Non-candidate | Solitary | 2022 | 5 | 0 |
| **JS15** | Male | Non-candidate | Solitary | 2015 | 5 | 0 |
| **JS16** | Female | Non-candidate | Solitary | 2015 | 4 | 0 |
| **JS17** | Male | Non-candidate | Solitary | 2022 | 4 | 0 |
| **JS18** | Male | Non-candidate | Solitary | 2018 | 5 | 0 |
| **JS19** | Female | Non-candidate | Solitary | 2018 | 7 | 0 |

Table S2. Pareto-smoothed importance sampling leave-one-out cross-validation (PSIS-LOO) diagnostics for Bayesian models of parasite diversity metrics in rehabilitating *N. javanicus*. The expected log predictive density (elpd_loo) summarizes predictive performance, p_loo represents the effective number of parameters, and LOOIC is the leave-one-out information criterion. Pareto-k diagnostics indicate that nearly all observations fell within the reliable range (k ≤ 0.7), suggesting stable cross-validation estimates.

| Model | elpd_loo (SE) | p_loo (SE) | LOOIC (SE) | k ≤ 0.7 | 0.7 < k ≤ 1 | k > 1 |
| --- | --- | --- | --- | --- | --- | --- |
| Shannon diversity | 24.36 (14.81) | 23.03 (4.89) | −48.71 (29.62) | 146 (99.3%) | 1 (0.7%) | 0 (0.0%) |
| Parasite richness | −164.97 (6.53) | 6.15 (0.43) | 329.95 (13.07) | 147 (100%) | 0 (0.0%) | 0 (0.0%) |

Table S3. Pareto-smoothed importance sampling leave-one-out cross-validation (PSIS-LOO) diagnostics for Bayesian Bernoulli mixed-effects models of parasite detection probability. The expected log predictive density (elpd_loo) summarizes model predictive performance, p_loo represents the effective number of parameters, and LOOIC is the leave-one-out information criterion. Pareto-k diagnostics indicate that the majority of observations fell within the reliable range (k ≤ 0.7), suggesting stable cross-validation estimates.

| Model | elpd_loo (SE) | p_loo (SE) | LOOIC (SE) | k ≤ 0.7 | 0.7 < k ≤ 1 | k > 1 |
| --- | --- | --- | --- | --- | --- | --- |
| Trichuris prevalence | −16.98 (3.45) | 5.19 (1.29) | 33.97 (6.91) | 146 (99.3%) | 1 (0.7%) | 0 (0.0%) |
| Strongylids prevalence | −41.27 (5.61) | 12.01 (2.25) | 82.55 (11.21) | 144 (98.0%) | 3 (2.0%) | 0 (0.0%) |
| Strongyloides prevalence | −78.95 (6.15) | 25.58 (2.61) | 157.89 (12.30) | 146 (99.3%) | 1 (0.7%) | 0 (0.0%) |
| Oxyurids prevalence | −28.84 (5.33) | 11.29 (2.64) | 57.69 (10.66) | 144 (98.0%) | 3 (2.0%) | 0 (0.0%) |

Table S4. Odds ratios (OR) and 95% credible intervals (CrI) for parasite detection probability models

| Predictor | *Trichuris* detection OR (95% CrI) | Strongylids detection OR (95% CrI) | *Strongyloides* detection OR (95% CrI) | Oxyurids detection OR (95% CrI) |
| --- | --- | --- | --- | --- |
| Status (non-candidate) | 0.91 (0.01–61.56) | 1.35 (0.03–55.15) | 1.73 (0.06–47.94) | 7.92 (0.13–436.84) |
| Sex (male) | 0.11 (0.00–4.71) | 0.28 (0.01–5.31) | 1.88 (0.20–17.46) | 2.94 (0.11–76.66) |
| Housing (solitary) | 0.24 (0.00–16.95) | 13.20 (0.29–613.11) | 0.26 (0.02–3.63) | 0.49 (0.01–27.11) |
| Rehabilitation duration | 4.90 (0.48–55.15) | 3.60 (0.55–30.57) | 3.67 (0.74–24.29) | 3.60 (0.42–35.16) |
| Weeks post-deworming | 0.69 (0.25–1.88) | 2.05 (1.03–4.53) | 2.59 (1.35–5.64) | 1.84 (0.83–4.66) |

Table S5. Pareto-smoothed importance sampling leave-one-out cross-validation (PSIS-LOO) diagnostics for Bayesian zero-inflated negative binomial models of parasite egg counts in rehabilitating *N. javanicus*. The expected log predictive density (elpd_loo) summarizes predictive accuracy, while p_loo represents the effective number of parameters. Pareto-k diagnostics indicate that the majority of observations fell within the reliable range (k ≤ 0.7), suggesting stable cross-validation estimates.

| Model | elpd_loo (SE) | p_loo (SE) | LOOIC (SE) | k ≤ 0.7 | 0.7 < k ≤ 1 | k > 1 |
| --- | --- | --- | --- | --- | --- | --- |
| Trichuris EPG | −104.46 (24.39) | 6.59 (2.09) | 208.92 (48.77) | 145 (98.6%) | 1 (0.7%) | 1 (0.7%) |
| Strongylids EPG | −206.11 (29.59) | 10.60 (2.08) | 412.22 (59.17) | 143 (97.3%) | 3 (2.0%) | 1 (0.7%) |
| Strongyloides EPG | −541.16 (39.08) | 20.11 (3.01) | 1082.33 (78.16) | 144 (98.0%) | 3 (2.0%) | 0 (0.0%) |
| Oxyurids EPG | −176.37 (28.56) | 8.60 (1.74) | 352.74 (57.12) | 142 (96.6%) | 5 (3.4%) | 0 (0.0%) |

Table S6. Individual-level gastrointestinal helminth infection summary for rehabilitating *Nycticebus javanicus*. For each individual, shown are release candidacy status, sex, housing condition, the number of fecal samples collected, cumulative parasite richness (the number of taxa ever detected across all samples), individual-level Shannon diversity (calculated from each individual's pooled egg counts), and the specific helminth taxa detected.

| ID | Status | Sex | housing | N  samples | Richness | Shannon  index | taxa_detected |
| --- | --- | --- | --- | --- | --- | --- | --- |
| JS01 | candidate | female | solitary | 14 | 1 | 0 | Strongylids |
| JS02 | candidate | female | group | 6 | 1 | 0 | *Strongyloides* |
| JS03 | candidate | female | solitary | 13 | 0 | 0 |  |
| JS04 | candidate | male | solitary | 12 | 1 | 0 | *Strongyloides* |
| JS05 | candidate | female | group | 8 | 1 | 0 | *Strongyloides* |
| JS06 | candidate | female | group | 11 | 3 | 1.05673 | *Trichuris, Strongyloides,* Oxyurids |
| JS07 | candidate | female | solitary | 11 | 1 | 0 | *Strongyloides* |
| JS08 | candidate | female | group | 7 | 1 | 0 | *Strongyloides* |
| JS09 | candidate | male | solitary | 13 | 2 | 0.53313 | Strongylids, *Strongyloides* |
| JS10 | candidate | male | group | 5 | 1 | 0 | *Strongyloides* |
| JS11 | noncandidate | female | solitary | 6 | 2 | 0.55807 | *Trichuris, Strongyloides* |
| JS12 | noncandidate | female | solitary | 5 | 4 | 0.674903 | *Trichuris*, Strongylids, *Strongyloides, Oxyurids* |
| JS13 | noncandidate | male | solitary | 6 | 2 | 0.414863 | *Strongyloides,* Oxyurids |
| JS14 | noncandidate | female | solitary | 5 | 1 | 0 | Strongylids |
| JS15 | noncandidate | male | solitary | 5 | 2 | 0.274243 | Strongylids, *Strongyloides* |
| JS16 | noncandidate | female | solitary | 4 | 3 | 0.983714 | Strongylids, *Strongyloides,* Oxyurids |
| JS17 | noncandidate | male | solitary | 4 | 2 | 0.573101 | *Strongyloides,* Oxyurids |
| JS18 | noncandidate | male | solitary | 5 | 2 | 0.692643 | *Strongyloides,* Oxyurids |
| JS19 | noncandidate | female | solitary | 7 | 2 | 0.268408 | Strongylids, *Strongyloides* |

Table S7. Published records of gastrointestinal helminths infecting slow lorises (*Nycticebus* spp.) across wild, captive, confiscated, and rehabilitation contexts. The table summarizes reported helminth taxa, host species, study location, and management context based on available literature and husbandry manuals. Records include both identified parasite species and higher taxonomic or unidentified groups, reflecting variation in diagnostic resolution among studies.

| Parasite Taxon | Parasite Species/Type | Host Species | Location/Study Type | Reference |
| --- | --- | --- | --- | --- |
| NEMATODA |  |  |  |  |
| Oxyuridae | *Lemuricola* (*Protenterobius*) *nycticebi* | *N. javanicus* | Wild, West Java, Indonesia | Rode-Margono et al. (2015) |
| Oxyuridae | *Lemuricola* (*Protenterobius*) *nycticebi* | *N. bengalensis* | Captive/confiscated, Bangladesh | Mondal et al. (2026) |
| Oxyuridae | *Oxyuris* spp. | *N. coucang* | Captive, YIARI, Indonesia | Ulfa (2014) |
| Oxyuridae | *Enterobius* spp. | *N. pygmaeus* | Captive, rescue center | Streicher (2004) cited in Rode-Margono et al. (2015) |
| Oxyuridae | *Enterobius* spp. | *N. pygmaeus* | Captive, Duke Primate Center, USA | San Diego Zoo Loris Husbandry Manual |
| Oxyuridae | Oxyurids (unidentified) | *N. coucang* | Captive, Duke Primate Center, USA | San Diego Zoo Loris Husbandry Manual |
| Oxyuridae | Oxyurids (unidentified) | *N. javanicus* | Captive, YIARI, Indonesia | Wibowo (2014) |
| Ascarididae | *Ascaris* spp. | *N. coucang* | Captive, YIARI, Indonesia | Ulfa (2014) |
| Ascarididae | *Ascaris* spp. | *N. javanicus* | Captive, YIARI, Indonesia | Wibowo (2014) |
| Ascarididae | *Ascaris* spp. | *N. coucang* | Captive, literature review | Setyorini & Werdateti (2005) |
| Ancylostomatidae | *Necator* spp. (hookworm) | *N. javanicus* | Wild, West Java, Indonesia | Rode-Margono et al. (2015) |
| Strongyloididae | *Strongyloides* spp. | *N. coucang* | Captive, YIARI, Indonesia | Ulfa (2014) |
| Strongyloididae | *Strongyloides* spp. | *N. javanicus* | Captive, YIARI, Indonesia | Wibowo (2014) |
| Strongyloididae | *Strongyloides* sp. | *N. menagensis* | Wild, Malaysian Borneo | Frias et al. (2018) |
| Strongyloididae | *Strongyloides* sp. | *N. coucang* | Captive, Duke Primate Center, USA | San Diego Zoo Loris Husbandry Manual |
| Strongylidae | Strongylids (unidentified) | *N. coucang* | Captive, YIARI, Indonesia | Ulfa (2014) |
| Strongylidae | Strongylids (unidentified) | *N. javanicus* | Captive, YIARI, Indonesia | Wibowo (2014) |
| Trichostrongylidae | *Trichostrongylus* spp. | *N. javanicus* | Wild, West Java, Indonesia | Rode-Margono et al. (2015) |
| Trichuridae | *Trichuris* spp. | *N. coucang* | Captive, YIARI, Indonesia | Ulfa (2014) |
| Trichuridae | *Trichuris* spp. | *N. pygmaeus* | Captive, San Diego Zoo, USA | San Diego Zoo Loris Husbandry Manual |
| Molineidae | *Pterygodermatites nycticebi* | *N. coucang* | Captive, San Diego Zoo, USA | San Diego Zoo Loris Husbandry Manual |
| Physalopteridae | *Physaloptera* sp. | *N. coucang* | Captive, Duke Primate Center, USA | San Diego Zoo Loris Husbandry Manual |
| Spiruridae (unidentified) | Nematodes (unidentified) | *N. coucang* | Captive, San Diego Zoo, USA | San Diego Zoo Loris Husbandry Manual |
| CESTODA |  |  |  |  |
| Hymenolepididae | *Hymenolepis* spp. | *N. coucang* | Captive, YIARI, Indonesia | Ulfa (2014) |
| Hymenolepididae | *Hymenolepis* sp. | *N. javanicus* | Captive, YIARI, Indonesia | Wibowo (2014) |
| Hymenolepididae | *Hymenolepis*-like ova | *N. pygmaeus* | Captive, San Diego Zoo, USA | San Diego Zoo Loris Husbandry Manual |
| Anoplocephalidae | Tapeworms (unidentified) | *N. coucang* | Captive, San Diego Zoo, USA | San Diego Zoo Loris Husbandry Manual |


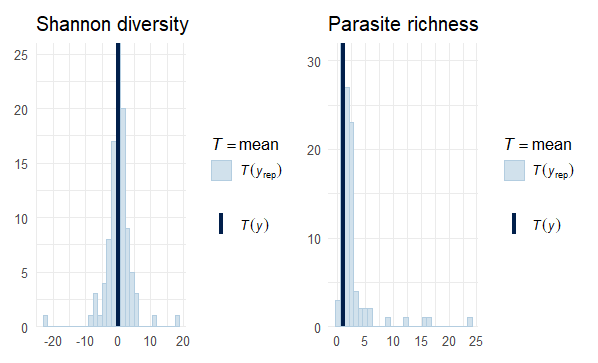


simulated mean Shannon diversity

simulated mean richness

count

count

Figure S1. Prior predictive checks for parasite Shannon diversity and richness models. Histograms show the distribution of simulated mean values under the prior predictive distribution; vertical bold lines indicate the observed mean. In both cases, the observed values fell within plausible prior predictive ranges.


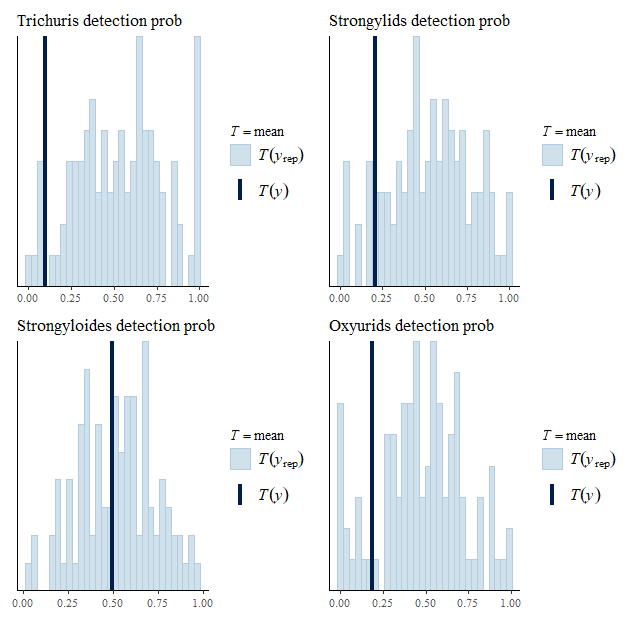


simulated mean detection probability

simulated mean detection probability

count

count


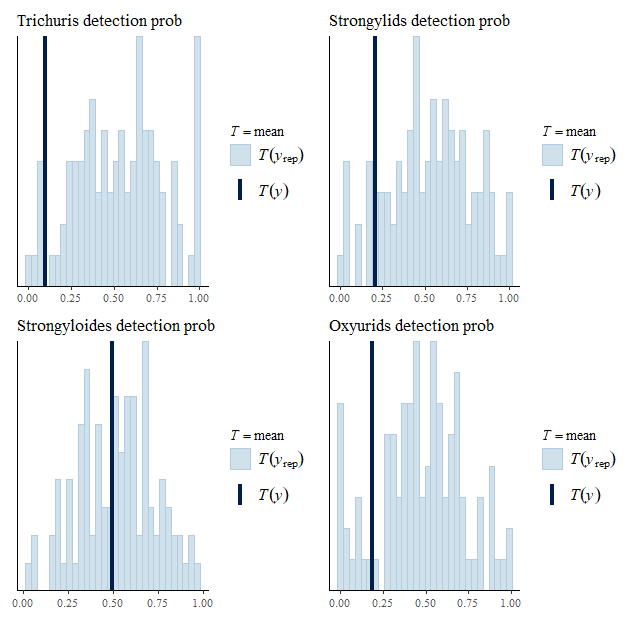


simulated mean detection probability

simulated mean detection probability

count

count

Figure S2. Prior predictive checks for parasite detection probability. Histograms show the distribution of simulated mean values under the prior predictive distribution; vertical bold lines indicate the observed mean. In all cases, the observed values fell within plausible prior predictive ranges.


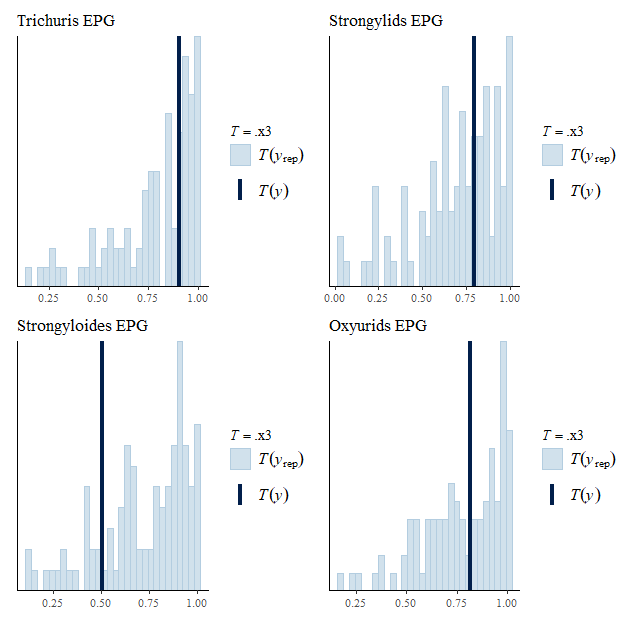

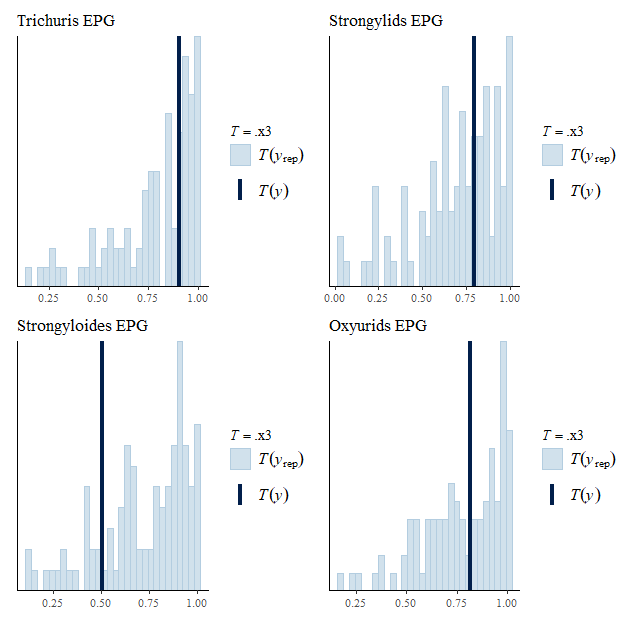


simulated mean EPG

simulated mean EPG

count

count

simulated mean EPG

simulated mean EPG

count

count

Figure S3. Prior predictive checks for parasite EPG models. Histograms show the distribution of simulated mean values under the prior predictive distribution; vertical bold lines indicate the observed mean. In all cases, the observed values fell within plausible prior predictive ranges.


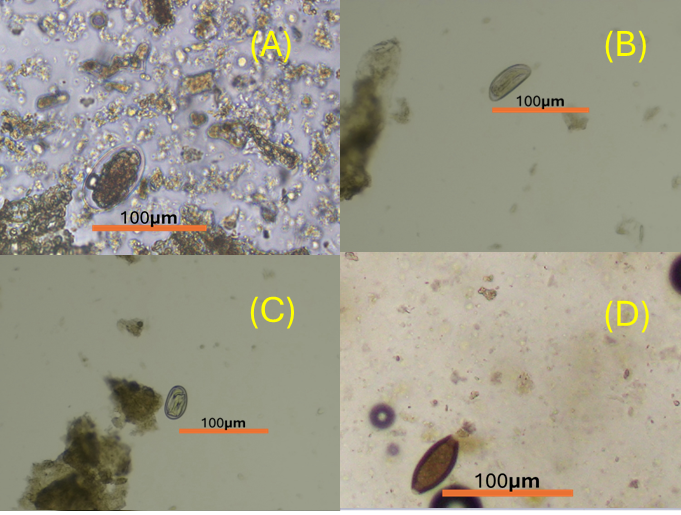


Figure S4. GI helminth eggs infecting rehabilitating *N. javanicus*: (A) Strongylids (B) Oxyurids, (C) *Strongyloides*, (D) *Trichuris*


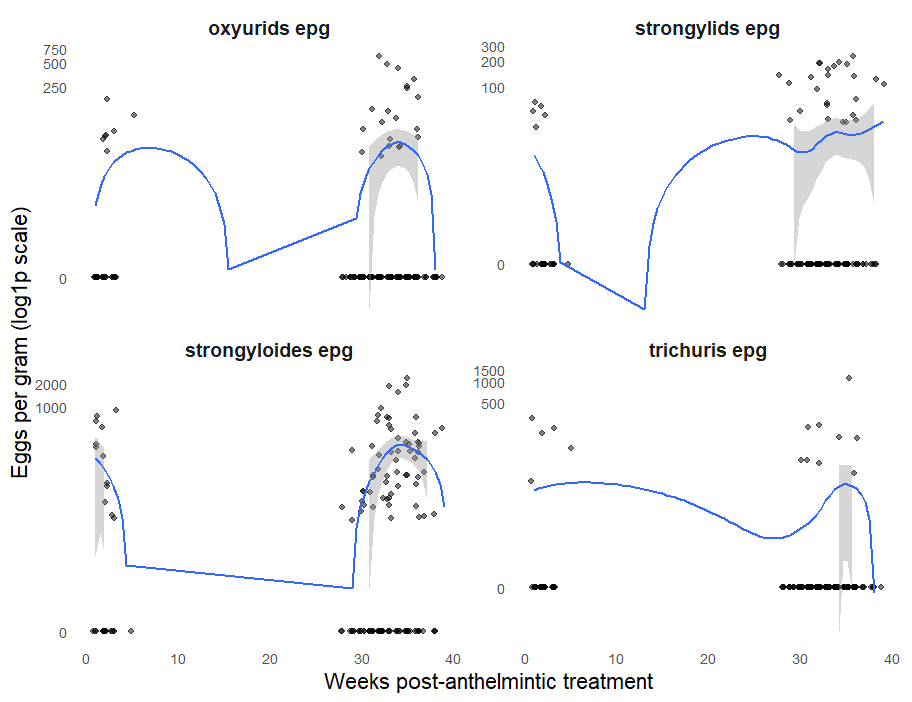


Figure S5. Density plot of parasite EPG related to weeks post-anthelmintic treatment

**References**

Fitch-Snyder H, Schulze H (eds), Larsen LC (compiler) (2003) San Diego Zoo Loris Husbandry Manual: Management of lorises in captivity. A husbandry manual for Asian lorises (*Nycticebus* & *Loris* ssp.). Last amendment 2 January 2003, San Diego Zoo Global, Escondido, CA, USA.

Frias, L., Stark, D.J., Lynn, M.S., Nathan, S.K.S.S., Goossens, B., Okamoto, M., MacIntosh, A.J.J., 2018. Lurking in the dark: cryptic Strongyloides in a Bornean slow loris. International Journal for Parasitology: Parasites and Wildlife. doi: 10.1016/j.ijppaw.2018.03.003

Mondal, J.K., Rabbi, M.R.R., Anjum, M., Chowdhury, M.F.S., Ahmed, R., Lamia, A.I., Hossain, M., Miti, A.A., Guala, C., Amin, I., Nobel, F., Rahman, S.C., Roos, C., Ahmed, T. (2026). New host and geographic record of Lemuricola nycticebi in the Bengal slow loris. Acta Parasitologica, 71(3), 108. doi:10.1007/s11686-026-01293-2

Rode-Margono, E.J., Albers, M., Wirdateti, W., Abinawanto, A., Nekaris, K.A., 2015. Gastrointestinal parasites and ectoparasites in wild Javan slow loris (*Nycticebus javanicus*), and implications for captivity and animal rescue. Journal of Zoo and Aquarium Research 3, 80-86. doi: https://doi.org/10.19227/jzar.v3i3.86

Setyorini, L., & Wirdateti. (2012). CACING PARASIT PADA *Nycticebus coucang*. Berkala Penelitian Hayati, 10(2), 93-96. https://doi.org/10.23869/436

Streicher U. (2004) Aspects of Ecology and Conservation of the Pygmy Loris N*ycticebus pygmaeus* in Vietnam. PhD thesis. Münich, Germany: Ludwig‐Maximilians‐Universität München

Ulfa N (2014) Kecacingan pada kukang Sumatera (*Nycticebus coucang*) di Pusat Rehabilitasi Satwa Primata Yayasan International Animal Rescue Indonesia (YIARI). Undergraduate thesis, Institut Pertanian Bogor

Wibowo MMA (2014) Kecacingan pada kukang Jawa (*Nycticebus javanicus*) di Pusat Rehabilitasi Satwa Primata Yayasan International Animal Rescue Indonesia (YIARI). Undergraduate thesis, Institut Pertanian Bogor
